# Biodiversity analysis in the digital era

**DOI:** 10.1098/rstb.2015.0337

**Published:** 2016-09-05

**Authors:** John La Salle, Kristen J. Williams, Craig Moritz

**Affiliations:** 1 Atlas of Living Australia, CSIRO National Research Collections Australia, GPO Box 1700, Canberra ACT 2601, Australia; 2 Land and Water, Commonwealth Scientific and Industrial Research Organisation (CSIRO), GPO Box 1600, Canberra ACT 2601, Australia; 3 Centre for Biodiversity Analysis and Research School of Biology, The Australian National University, Acton ACT 2601, Australia

**Keywords:** biodiversity informatics, e-research infrastructure, evolution, biogeography, environment

## Abstract

This paper explores what the virtual biodiversity e-infrastructure will look like as it takes advantage of advances in ‘Big Data’ biodiversity informatics and e-research infrastructure, which allow integration of various taxon-level data types (genome, morphology, distribution and species interactions) within a phylogenetic and environmental framework. By overcoming the data scaling problem in ecology, this integrative framework will provide richer information and fast learning to enable a deeper understanding of biodiversity evolution and dynamics in a rapidly changing world. The Atlas of Living Australia is used as one example of the advantages of progressing towards this future. Living in this future will require the adoption of new ways of integrating scientific knowledge into societal decision making.

This article is part of the themed issue ‘From DNA barcodes to biomes’.

## 1. Introduction

There is an urgent need to document and understand nature at a rate that will provide us with an informed system-level response to the accelerating impacts that humans are having on the world. Major challenges will include food security, emerging diseases, managing natural and agricultural landscapes in a sustainable fashion and interactions with invasive species (native and alien); coinciding with an era of rapid environmental change [1]. This urgency is evident at an international level, given the importance of data to achieving the goals of the Convention on Biological Diversity, expressed through the Aichi Biodiversity Targets for 2020 and initiatives such as the Intergovernmental Platform for Biodiversity and Ecosystem Services (IPBES) and research consortiums such as GEO BON (Group on Earth Observations Biodiversity Observation Network) [2,3]. Essential biodiversity variables (EBVs)—a minimum set of essential measurements for studying, monitoring and reporting on biodiversity and ecosystem change—are proposed as one mechanism for addressing this goal [4], although practical implementation remains a challenge [5,6]. In this context, maintaining investment in biodiversity surveys and descriptions, including novel applications of predictive spatial modelling, increased efficiency of phenotyping and (meta)genomics are key. Without strong, ongoing support for data gap identification, generation and curation, the most advanced informatics will be an empty shell.

Bringing biodiversity analysis into the digital world will provide all people and jurisdictions with easy and rapid access to the authoritative and comprehensive evidence and knowledge that they need to make informed decisions. Advances in biodiversity informatics, computer technology and governance structures allow information to be shared and processed at unprecedented speed, creating an environment to enable truly rapid biodiversity analysis [7,8]. As data, information and knowledge become accessible, available and able to be analysed in new and different ways, new uses for (and value of) that information are continually being discovered and will increase our ability to inform research and policy [9]. Web-based e-infrastructure will take advantage of, and further enable, the increasing involvement of citizen scientists in supporting ecological and environmental research [10,11].

It is essential that the biodiversity analysis laboratory of the future can integrate a variety of taxon-level data types (e.g. distribution, genome, phenome, traits and species interactions) and enable analysis of that information in an evolutionary and environmental framework to produce more comprehensive understanding of the drivers of biodiversity and the potential impact of global change on biodiversity [12]. This achievement will necessarily require changing some of the norms of scientific endeavour to allow a new generation of digital scientists running ‘Big Data’ experiments to push the boundaries and transform knowledge of ecology [13].

This paper takes a wide view of biodiversity analysis. Well-governed interoperable e-infrastructure and work flows should support biodiversity discovery and documentation, environmental monitoring, reporting and decision making, as well as the capacity to run fundamental scientific modelling experiments to build understanding of biodiversity evolution, biogeography and dynamics in a changing world [14,15].

For the most part, the key components of this e-research infrastructure already exist. The digital transformation is providing a suite of emerging and disruptive technologies, which are changing the way we think about managing, discovering and delivering biodiversity and environmental data [16]. These have been embraced by a wide range of global initiatives, which are producing remarkable results for specific elements of biodiversity information (e.g. spatially explicit distributional data, species trait and other profile information). It is now time to coordinate the sharing of information in an integrated way to facilitate rapid biodiversity analysis, novel knowledge and its timely use in decisions [5,13]. Although this outcome may appear challenging on a global scale, the Atlas of Living Australia (ALA, www.ala.org.au) shows that such a digital platform for knowledge sharing can be created on a continental/national scale [17]. Examples of outcomes from this integration are used to illustrate the benefits of such e-infrastructure, although global level implementation will require coordination of both e-infrastructure efforts and data standards [5,7,18,19].

## 2. Key components of a virtual biodiversity analysis e-research infrastructure

Truly integrated biodiversity e-infrastructure will bring together computable data about taxa which, when placed in an environmental and evolutionary context, will enable rapid biodiversity analysis and facilitate informed decision-making. As important as the data and analysis tools are, so too is improved capacity to visualize and share the knowledge derived from these analyses with a broad audience. Finally, we acknowledge the need for data aggregators and servers to strive to develop tools to enable data quality to be improved at source, such as by the natural history collections that curate the original data [20].

Key elements in this web-based e-infrastructure include taxon-level information, environmental and other contextual layers, the ability to incorporate evolutionary and functional perspectives, informatics and analysis tools supporting applications, all of which must operate under an agreed set of principles promoting data discovery and sharing, open infrastructure and collaboration (table 1).

**Table 1.** Core principles to support e-research infrastructure for biodiversity knowledge generation.

type	statement of intent
collaboration	we must develop an inclusive model for participation by all stakeholders, from local to national levels, in biodiversity information
sharing	we must adopt procedures to prevent duplication of effort, build on past investments and create shared efficiencies to the greater benefit of all
science	we must organize data to provide the best possible sustainable support for excellent, independent research, now and in the future
learning	we must enable novel or alternative approaches to new knowledge generation to be explored
integration	we must be able to bring different types of data into a shared environment
quality	we must enable users to understand the level of evidence and authority for all data elements and have services to help improve data quality at source
open access	we must promote and facilitate free and open use of data—and infrastructure
acknowledgement	we must create an environment where individual and collective endeavours can be recognized and built upon
delivery	we must provide comprehensive, stable, authoritative services that meet the needs of stakeholder groups
innovation	we must establish a model for continuous modernization and improvement of services. Open infrastructure will support innovative new uses of infrastructure and data
collect data once—make it freely accessible—use it many times	

### (a) Taxon information

Incorporating a range of taxon-level attributes will enhance efforts informing effective management of sustainable environments into the future. In particular, we need data systems that enable us to move from ‘what is where’ questions to ‘why is it there’, ‘what does it do’ and ‘what can we do about it’. A list of the types of taxon data that we should be able to integrate in an e-research environment would include the following:

#### (i) Distribution

Spatially explicit biodiversity data for taxa are the mainstay of many biodiversity analyses and provide a form of computable data that enable a great many uses. The Global Biodiversity Information Facility (GBIF—www.gbif.org) currently aggregates and provides over 577 million occurrence records (October 2015), and their science review [21] provides numerous examples and over 200 references to the use of GBIF mediated occurrence data to support research activities in the areas of invasive alien species, impacts of climate change, species conservation and protected areas, biodiversity and human health, food, farming and biofuels, ecosystem services and advancing biodiversity science. An example of e-infrastructure that leverages GBIF records is Map of Life (www.mol.org), which connects spatially explicit point data with layers of expert geographical ranges, conservation reserves and values of evolutionary distinctness and IUCN (International Union for the Conservation of Nature) status [22].

#### (ii) Genetic/genomic information

Over recent decades, various DNA barcoding initiatives have yielded broad-scale coverage of species and continents for a few standardized reference genes. Hebert *et al.* [23] showed that continent-wide DNA barcode libraries (and by extension, other types of genomic information) could be generated quite rapidly through targeting well-curated and identified material in natural history collections to link sequence records to authoritative voucher specimens. Now, with the capacity to efficiently generate sequence data for hundreds to thousands of genes from populations to entire clades [24,25], we are set to transform molecular systematics yet again. Further, the burgeoning field of environmental genomics—including metabarcoding and metagenomics—will add yet more capacity for biodiversity analyses and monitoring [13,14]. It may not be practical to combine all these types of information within a single e-infrastructure in the near future; however, discovery of relevant data across platforms can be enabled through use of uniform metadata standards and the ability to import molecular analysis products (e.g. phylogenetic trees and trait suites; see the following sections).

#### (iii) Genome to phenome

To move from mapping diversity to understanding how it evolved and functions, it is imperative that we combine distribution data with a range of genomic and phenomic data. Integrating genetic and morphological attributes, as well as other forms of trait data such as behaviour, life history and chemical composition and gene expression, informs and improves species discrimination, taxonomy, phylogenetic analysis and a range of other biodiversity data integration applications [26].

Differences in data types and standards have hindered the ability to bring all these types of ancillary data into a single analysis platform. Researchers often adopt short-term individual approaches to solve a data integration problem to meet their analysis requirements. These are key challenges that will need to be addressed to create the e-infrastructure necessary for collaborative, comprehensive and efficient biodiversity analysis.

#### (iv) Trait data

There are a variety of forms of data that can be considered as species traits, including morphology, chemical, habitat and life history characters. One important set is morphological characters, and there needs to be the ability to capture geo-referenced character information in a fashion that enables understanding of variation within and between species and provides sets of characters that can be used (and re-used) in identification keys and phylogenetic, evolutionary and macroecological analyses [27,28]. Global examples of trait banks include the Encyclopedia of Life Trait Bank (www.eol.org/traitbank) [29] that delivers 11 million records for over 330 attributes for 1.7 million taxa, and the TRY Plant Trait Database (www.try-db.org) [30] that delivers 5.6 million trait records from 100 000 plant species.

Image libraries are a way of depicting morphological characters (as well as spatial distribution of characters) and images can come in a variety of forms: specimen images, scanning electron micrographs, CT/MicroCT scans [31,32], three-dimensional images [33,34] and whole drawer images [35]. However, image libraries are only a starting point and there remains the need to extract character information from them in such a way that the information can be shared, made freely available and re-used. Methods to extract information might include experts, crowdsourcing through digitization portals [36] or even automated extraction by machines [37].

#### (v) Species/trophic interactions

Interactions between species are key components of maintaining ecosystem stability and are central to the diversification and organization of life [38]. Global environmental change can produce adverse impacts on species interactions to the detriment of ecosystem stability [39]; thus, being able to record and track species interactions can inform policy, operational and research direction. In the simplest form, a single species interaction could be recorded as a species trait; however, complex food webs are common in nature, contain multiple interactions and are living laboratories for ecological research [40,41]. Clearly, the ability to convey this information as an integrated component of a future biodiversity-analysis laboratory will have immense value, but will require some quite sophisticated infrastructure. GloBI (Global Biotic Interactions: www.globalbioticinteractions.org/about.html) [42] is an example of such a database, delivering over 1.3 million interactions for 113 000 distinct taxa.

### (b) Taxonomic framework

Any attempt at documenting biodiversity has to be placed in a taxonomic framework to give it credibility and ensure that information can be universally shared and associated with the correct taxon. A ‘standard’ taxonomic framework might consist of scientific names, species concepts and classification.

There is a great deal of complexity with handling names, including synonymy, homonymy, misidentifications and a variety of common names in use for any given organism. Life science identifiers (LSIDs) are unique identifiers that could be applied to each name, or species concept, to avoid confusion and ensure stability [43].

Species concepts are biological concepts, fluid and often subjective in interpretation [44]. Each species concept will encompass one-to-many scientific names, with one being the senior, valid name, although there can be disagreement on which name or combination to use. Classifications arrange species into higher taxa, such as genera, subfamilies and families. They can be even more subjective than species concepts, with often several different classifications being used at any given time. A modern informatics infrastructure must be able to display differing views of species and higher classifications to be of optimal value to the user community.

In addition to a standard Linnaean taxonomic framework, a variety of biodiversity analyses might need the use of interim taxonomic nomenclature. Operational taxonomic units (OTUs) [45] might represent candidate species that can be recognized (morphologically or genetically) but are yet to be formally named. An example of a DNA-based delineation would be a barcode index number (BIN) [46]. It is important to be able to integrate information associated with BINs with information about closely related species that have been formally named (and that may also have an associated BIN).

There must also be the ability to import a list of (often intraspecific) OTUs along with associated data (genomic, trait and distribution) for analyses within the e-research laboratory, even if this information does not (yet) have a persistent home within the research infrastructure. To address this need and the fluidity of species and higher taxon concepts, there is value in representing current knowledge via the phylogenetic trees below and above the species level. Analyses of spatial genetic diversity within described species—phylogeography—frequently reveal high levels of lineage diversity that often remains invisible to taxonomy, and hence, inaccessible to most data infrastructure. Yet, using phylogenetic representations of diversity, this rich source of information can be effectively visualized for scientific and conservation purposes (e.g. [47]).

### (c) Phylogeny

**Figure 1.**
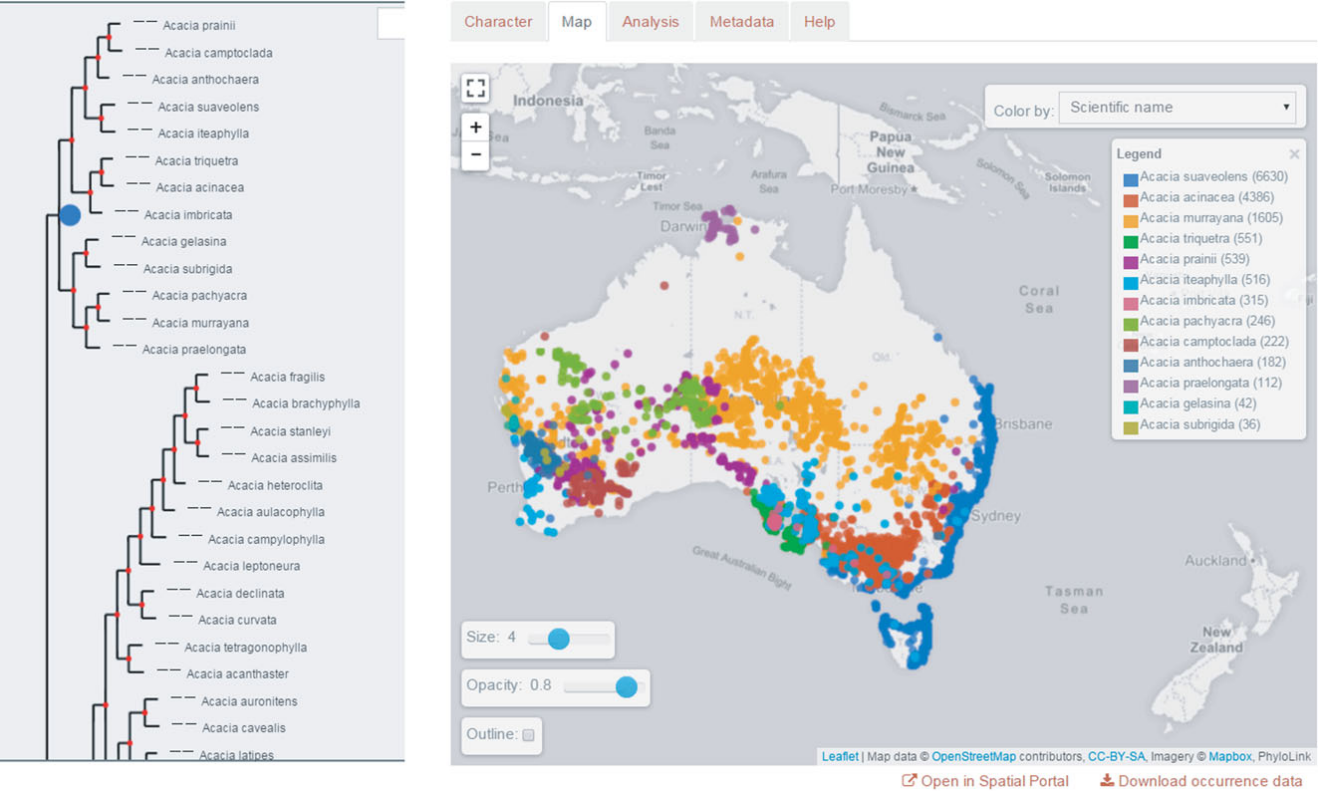
The ALA's phylogenetics tool integrates phylogenetic trees and spatial mapping so that phylogenies can be represented spatially, for example by species occurrence or character. Here, the occurrence of *Acacia* species from the clade highlighted by the blue node to the left is mapped and coloured by species.

Adding a phylogenetic component to biodiversity informatics is crucial to understanding how evolutionary responses to past environmental change have shaped current biodiversity. A phylogenetic framework for the biodiversity analysis laboratory allows us to develop new tools to integrate and analyse big data across taxa, regions and timescales. The results will yield unparalleled understanding of the distribution of genetic, taxonomic and functional diversity over space and time. In turn, this will provide novel insights into the potential futures of biodiversity and enhance strategies to protect it [28,48,49]. This will serve to bridge the current void between conservation policy and practice by showing how knowledge of evolutionary processes can improve large-scale planning, and it will deploy this know-how to predict and improve management of biodiversity. Initial efforts towards this are being developed in the ALA (http://phylolink.ala.org.au/) and allow for the import of phylogenetic trees into the Atlas e-infrastructure environment where they can be combined with mapping and analysis tools and contextual layers [50,51] (figure 1).

### (d) Environmental information

Environmental information is fundamental to understanding in ecology. A wide variety of environmental factors play a role in the distribution, health and maintenance of biodiversity. The ability to analyse spatially explicit and temporally varying biodiversity data in respect to these environmental (or other contextual) variables provides tremendous power to the study of biodiversity and predictive analyses based on biodiversity data. New initiatives in environmental modelling and remote sensing are rapidly advancing the spatial and temporal resolution and three-dimensional detail by which environmental attributes such as soil can be mapped [52,53], with potential to overwhelm storage and analysis capacity of e-infrastructures. Yet this example belies the general dearth of information on edaphological data and the multiple layers of missing habitat information, requiring concerted efforts to bring together and harmonize relevant data regionally and globally. The digital laboratory of the future will need to provide guidance and a portal to an array of environmental variables of potential relevance in biodiversity research—helping integrate knowledge across realms, from terrestrial and freshwater to coastal and marine toward a realization of the digital Earth concept [54,55].

Finding and organizing the diverse sources of spatial environmental data needed for biodiversity analysis is not trivial [17]. The ALA started tackling this problem in 2010. The Spatial Portal [56] (http://spatial.ala.org.au/) presently integrates multiple types of biological data (collection records, survey observations, checklists and range maps) with over 450 remotely derived abiotic and biotic contextual layers (such as climate, terrain, soils, vegetation, land cover, land use, jurisdiction boundaries) and is reaching capacity using current technology. A new distributed approach is needed, one that supports discovery, access and manipulation of data to derive biologically meaningful predictor variables [57]. DataOne is an example of collaborative e-infrastructure heading in this direction [55] and in this context, the concept of a KLAS—knowledge, learning and analysis system—is visionary [13]. The BCCVL (Biodiversity and Climate Change Virtual Laboratory) [58] provides a sandpit of environment variables for analysis that are pre-selected from easily accessed sources and can be easily updated when appropriate. New spatial analysis technologies and information standards may in the future negate the need to locally aggregate spatial data in a common format like a cube and instead allow flexible interrogation at source via Web services, combined in a model, with predictions visually represented at appropriate resolutions. Open geospatial data standards and application programming interfaces (APIs) are essential to this vision of interoperability, with the Open Geospatial Consortium leading on these developments [59].

Furthermore, there are some significant initiatives underway that we would want a virtual biodiversity e-infrastructure to connect to in due course. It will be critical to engage with communities of practice such as the OBO (Open Biomedical Ontologies) ontology foundry—a collective of developers committed to interoperable ontologies (common controlled vocabularies) that are both logically well formed and scientifically accurate [60]. The wider biodiversity data community will need to increase its awareness of, for example, existing ontologies for contextualizing biological entities such as ENVO (Environmental Ontology; http://www.environmentontology.org/) [61] and avoid ‘silos and reinvented wheels’ [62] by adopting shared principles (e.g. http://www.obofoundry.org/) and participate in established networks (e.g. see resources and projects on http://bioportal.bioontology.org). The Ocean Data Interoperability Platform (ODIP) is an example of a community of practice developing a common framework for marine data management. ODIP's initial focus on cruise information is now extending to observation data [63]. Other initiatives to be aware of that may soon interface with the biodiversity science community or provide examples of how to advance collaborative infrastructures are the US-based EarthCube, http://earthcube.org/ (mostly solid Earth sciences) and the community effort called Earth Science Information Partners (ESIP). EarthCube is considering ‘Collaborative Resource Incubators’ to increase science community-driven innovation for infrastructure solutions [64]. The Research Data Alliance, which promotes open sharing of data (https://rd-alliance.org/), and INSPIRE—Spatial Information in the European Community (http://inspire.ec.europa.eu/)—and the common principles upon which they are founded are also relevant.

An important emerging project for the biodiversity community is GLOBIS-B (http://www.globis-b.eu), which aims to foster global cooperation of biodiversity research infrastructures and biodiversity scientists to advance the implementation and calculation of EBVs [5]. GLOBIS-B (GLOBal Infrastructures for Supporting Biodiversity research) builds on the roadmap for interoperability developed by the preceding CReATIVE-B project (Coordination of Research e-infrastructures Activities Toward an International Virtual Environment for Biodiversity, 2011–2014). Another EU initiative is LifeWatch (http://www.lifewatch.eu/)—European infrastructure for biodiversity and ecosystem research—aimed at providing researchers with access to virtual laboratories of biodiversity data with advanced biodiversity-informatics tools [65]. A challenge for the future will be addressing global interoperability among the different architectures across a rapidly emerging plethora of eResearch platforms.

### (e) Tools

**Figure 2.**
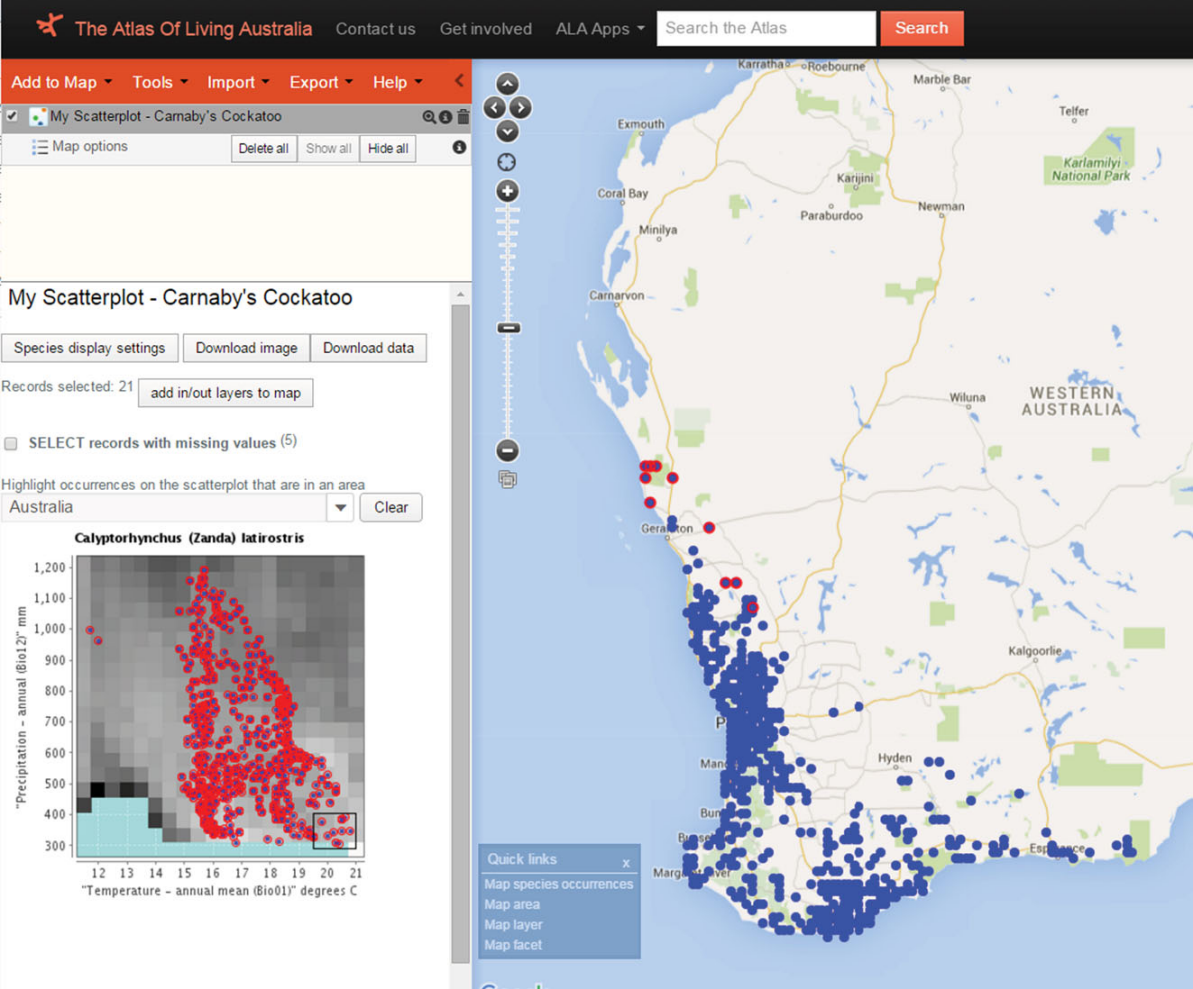
The ALA scatterplot analysis maps distribution points (right) in two-dimensional environmental space; here, we show a grid of rainfall versus temperature (left). Placing the small box around the ‘hottest, driest’ points on the left produces the red circles for those points on the distribution map (for advanced examples see http://www.ala.org.au/spatial-portal-help/scatterplot/). The ‘cool, wet’ outliers on the plot are spurious locations in eastern Australia where the species does not occur naturally.

**Figure 3.**
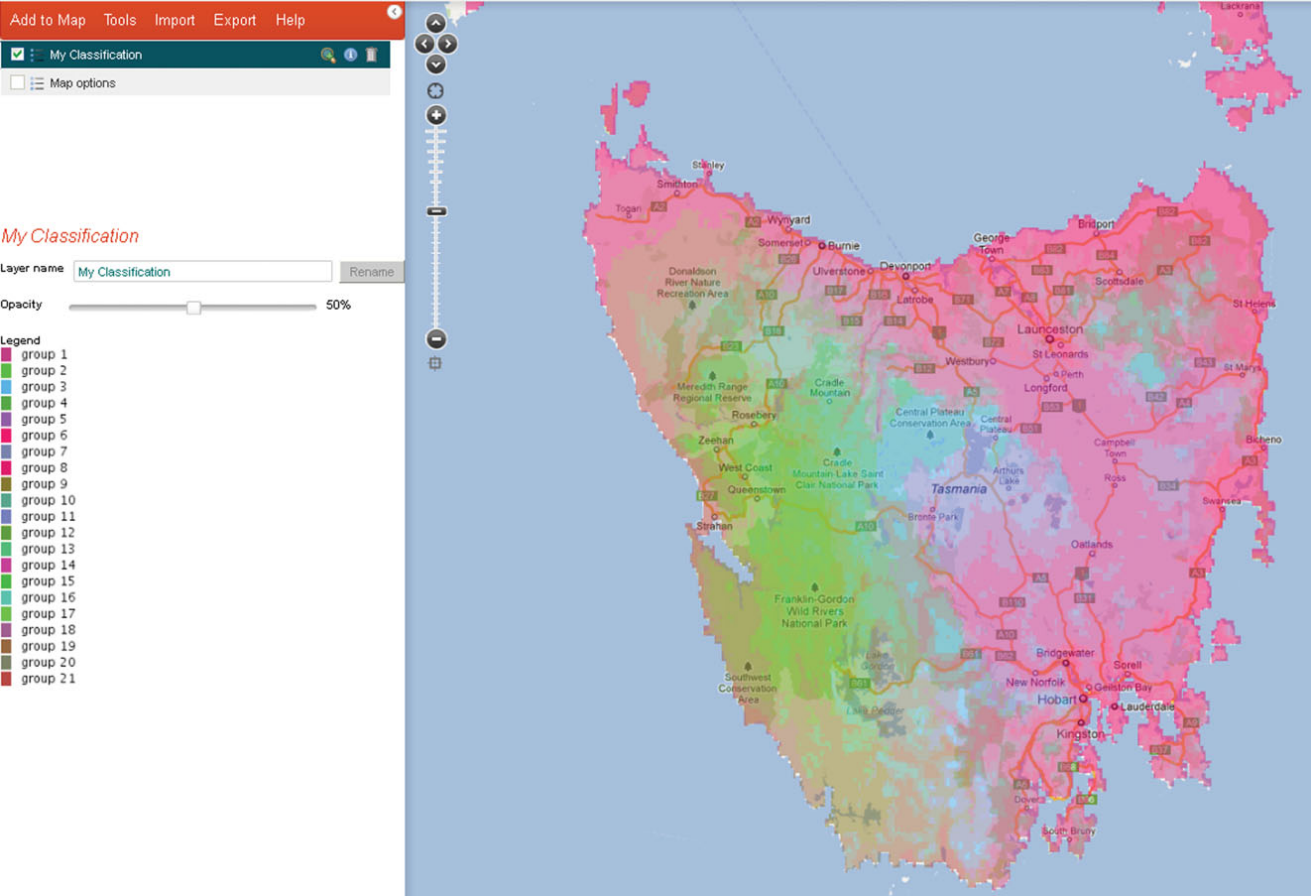
The ALA classify tool enables a selection of (ideally) relatively uncorrelated environmental layers for a predefined area to be classified into characteristic domains for a given number of groups, shown here for Tasmania—a large continental island off south-eastern Australia (image credit: http://www.ala.org.au/spatial-portal-help/classify/). The classification uses the ALOC algorithm [66].

An e-research environment should include tools for data discovery, access, integration, filtering, visualization, analysis, mapping and annotation. Currently, the spatial portal within the ALA links biological and environmental data to a limited suite of visualization and modelling tools—ranging from simple graphing and tabulation functions (e.g. figure 2) through to ecological classification (e.g. figure 3), species- and community-level biodiversity modelling techniques [17]. These tools enable a variety of exploratory analyses and assessments, including predicting threatened species ranges and/or helping to identify species climatic requirements [57,67,68]. This open software architecture, including a standard set of tools embedded in the spatial analysis portal, is being adopted by other countries (e.g. Atlas of Living Scotland, http://www.als.scot/).

There is a balance between creating a wide range of specific tools for biodiversity analysis and giving users the ability to develop or adapt their own tools. To facilitate user flexibility, the ALA supported the development of an R programming language package for researchers (ALA4R) [69] enabling direct access to hosted data resources using APIs. Perhaps, one critical tool missing from the ALA platform is a means of routinely identifying gaps in biological data collections using multivariate environmental space [70,71]. An early Web-based implementation of the survey gap-analysis method using the environmental diversity concept [72], under the auspices of GBIF [73], is no longer available. The addition of this tool is one example of an easily achieved task with high added value to support biodiversity discovery and data acquisition priorities.

Data availability and quality are important aspects of e-infrastructure, which must enable data capture, discovery, visualization and analysis for a range of purposes—not all of which are known at the outset. It will be equally important to develop sets of tools and services for data checking and revision, with feedback mechanisms between data custodians/providers and users, to capture their annotations about data quality and improve fitness for use for all practitioners [74]. As one example, VertNET (www.vertnet.org), an aggregated database of museum records for vertebrate species, enables users to submit annotation on individual records directly to the contributing collection, thereby correcting errors at the source [20].

It is important to note however, that there have been significant architectural shifts in recent years, which could challenge some of the existing biodiversity infrastructures. E-research will increasingly depend on Web-architectures with persistent URIs (uniform resource identifiers) being the default expectation by which data are linked. A URI is a string of characters to identify a name or a Web resource and can be classified as locators (URLs), as names (Uniform Resource Names—URNs) or as both. LSIDs are represented as URNs—for example, see [75]. The exact location of a URN may change, but the owner of the URN can expect that the resource can always be able found somehow. There is also a shift towards URI-based APIs, rather than query-based services (this is referred to as REST). Representational state transfer (REST) is a set of software architecture principles [76] that have become the default for most Web and mobile apps. Web service APIs that adhere to the REST architectural constraints are called RESTful APIs and allow higher-performing, more easily maintained software for Web services [77].

## 3. Discussion

### (a) Benefits

The wish list for e-infrastructure outlined in this paper is not an end in itself. It is needed to inform a range of outcomes, including conservation, environmental monitoring and reporting, area management, ecosystem modelling, sustainable food and health, biosecurity, biodiversity discovery and documentation, as well as supporting community engagement and research across a range of biodiversity science activities.

As mentioned above, access to GBIF-mediated spatially explicit biodiversity data resulted in over 200 publications across a range of activities [21] and these are in addition to grey literature, government reporting and directly informing environmental management decisions and policy. Adding an environmental and/or evolutionary context expands the usage of cases to include a much wider range of activities within a single environment, such as developing sustainable revegetation strategies under climate change [67], understanding climatic envelopes and adaptability of tree species [68], understanding environmental variables for biodiversity modelling [57] or predicting the evolution of tolerance to other environmental factors such as salinity [78].

Such a digital infrastructure will see gains in efficiencies by greatly reducing the amount of time necessary to perform biodiversity analysis, meaning that we can respond to threats to ecosystems and biodiversity in a much more meaningful time frame [13,79]. As much as 90% of a research project can be in data discovery, collation and integration. Effective e-research infrastructure means that the majority of research time is spent on research [58].

Experience from the ALA demonstrates that provision of robust and open infrastructure with Web services enables a variety of activities. For example, both the Biodiversity and Climate Change Virtual Laboratory (www.bccvl.org.au) [58] and Edgar (http://spatialecology.jcu.edu.au/Edgar/) are separate ventures that draw in ALA data to support analyses of the impact of climate change on biodiversity.

### (b) Future opportunities

Presently, there is no single e-research infrastructure that provides all the components described in this paper. At the global level, many of the data types mentioned are handled by separate initiatives, and the list of these initiatives provides examples of forward vision and advanced biodiversity informatics capabilities. These include (as a mere sample and with apologies for omissions): the Global Biodiversity Information Facility (GBIF—www.gbif.org), the Encyclopedia of Life (EOL—www.eol.org), Catalogue of Life (www.catalogueoflife.org), Map of Life (www.mappinglife.org), the International Barcode of Life (iBOL—www.ibol.org), Genbank (www.ncbi.nlm.nih.gov/genbank/), Open Tree of Life (http://opentreeoflife.org/) and iDigBio (www.idigbio.org). It is clear that any future model for biodiversity infrastructure must build on the strengths and collaboration of these global initiatives rather than try to duplicate or replace them. However, now there is a need for these initiatives to provide a clear vision and strategy as to how they will work together to create true global infrastructure, which is bringing together (and building on) the current capabilities to deliver integrated biodiversity information in a seamless manner. Ensuring data consistency in this landscape will allow big data biodiversity analytics to inform all aspects of biodiversity analysis and assessment to provide an informed response to global change.

Meaningful thinking about the future of biodiversity analysis needs to go past a discussion of current technology and platforms and focus on what we need to achieve to attain the environmental sustainability necessary for our future. This means identifying major gaps in e-infrastructure, agreeing on a set of priority goals and working together to accomplish them.

We must create an order of magnitude increase in the rate at which we capture biological and environmental data. This means that biologists across a range of disciplines can no longer justify non-digital data capture. ‘Born digital’ data will come from field observations as well as imaging biological collections, which are repositories of big data and hold longitudinal data through time that cannot be found anywhere else. We need to embrace a range of computer vision, machine learning and remote sensing techniques as well as robotics platforms to achieve our goals [80]. Crowdsourcing of data capture is an increasingly viable option, and we have to work with citizen science communities to enable the process and provide feedback to continually improve the quality of our citizen scientists and the data they produce.

We have to provide an infrastructure framework for managing these data in a way that they can be mobilized, discovered, searched, integrated and analysed and made freely and openly available to the wider research and policy community. The community has to come together to develop this vision and sell it with a common voice, as highlighted by the Belmont Forum's survey on open data [81]. We can no longer afford to have informatics initiatives that do not use Web services to share data, services and analysis tools, or that want to do everything themselves and duplicate scarce resources in their efforts.

We cannot hold on to technology. The rate of technological advance is so rapid that anything that we are planning or doing today will be out of date in 5 years. However, the drivers for what we need to accomplish will remain the same, so we have to embrace emerging technology and update our thinking as we go.

The already overstretched taxonomy community has to invent new practices and norms that will allow a step increase in the rate of species discovery and description [82,83]. An inventory of life on Earth is critical to environmental management, yet we are centuries away from achieving this at our current rate of progress. To date we have described something close to two million species. What will it take to describe the next million in 10 years? That is the rate of species discovery that is consistent with the needs we are facing and it will require some radical changes in thinking and work practices.

And finally, we must remember that the largest impediments we will face in creating true global infrastructure are not technical. We need to encourage workers to welcome and use open data, open infrastructure and services, and shared, virtual environments to truly accelerate biodiversity discovery and documentation to the level at which it can support timely and meaningful responses to the global challenges we will be facing.
